# Electrocorticography During Deep Brain Stimulation Surgery for Movement Disorders: Single-Center Experience

**DOI:** 10.3390/brainsci16060561

**Published:** 2026-05-26

**Authors:** Helena Ljulj, Kurt Lehner, Kimberley Wyse-Sookoo, Toren Arginteanu, Kelly A. Mills, Yousef Salimpour, William S. Anderson

**Affiliations:** 1 Department of Neurosurgery, Johns Hopkins School of Medicine, Baltimore, MD 21287, USA; helenljulj@gmail.com (H.L.); klehner3@jhmi.edu (K.L.); targint1@jhmi.edu (T.A.);; 2 Department of Neurology, Johns Hopkins School of Medicine, Baltimore, MD 21287, USA; kwyseso1@jh.edu (K.W.-S.);; 3 Department of Biomedical Engineering, Johns Hopkins School of Medicine, Baltimore, MD 21218, USA

**Keywords:** deep brain stimulation, electrocorticography, movement disorders, Parkinson disease

## Abstract

**Highlights:**

**What are the main findings?**

- High-density intraoperative ECoG during awake DBS surgery was feasible in patients with Parkinson’s disease and essential tremor.
- In this 36-patient single-center series, postoperative complications were limited, and overall rates were comparable to or lower than standard DBS surgery.

**What are the implications of the main findings?**

- Standardized high-density ECoG placement through the DBS burr hole provides a practical platform for studying cortical physiology in movement disorders.
- This approach may support biomarker discovery and the development of adaptive closed-loop neuromodulation systems that could improve future treatment strategies for Parkinson’s disease and essential tremor.

**Abstract:**

**Objective:** Electrocorticography can serve as an intraoperative research tool during deep brain stimulation procedure, when patients are awake to participate in behavioral tasks or to allow recordings while awake but at rest. This report aims to describe the electrocorticography methods used in awake patients undergoing deep brain stimulation surgery at a single center and to describe the feasibility, safety, and usefulness of high-density electrocorticography for capturing high-resolution neurophysiological data during deep brain stimulation surgery. We hypothesize that the use of high-density electrocorticography and multi-subject integration of cortical data enables improved spatial resolution and data analysis compared to prior studies employing lower-density electrodes and primarily single-subject analyses. **Methods:** Data were obtained from patients undergoing awake deep brain stimulation surgery for the treatment of Parkinson’s disease or essential tremor at Johns Hopkins Hospital between March 2022 and September 2024. Electrophysiological and anatomical data were analyzed, with localization in the anterior commissure and posterior commissure and Montreal Neurological Institute coordinate systems. Surgical complications were monitored for at least six months postoperatively. **Results:** Thirty-six patients (26 with Parkinson’s disease, 10 with essential tremor) were enrolled in the study. In one case, anatomical placement was inadequate for neurophysiological analysis. Postoperative complications included three infections (8.3%) and one chronic subdural hematoma (2.8%), with no permanent neurological deficits. Observed complication rates were within the range reported in the literature for standard deep brain stimulation surgeries without electrocorticography. Anatomical and neurophysiological analysis demonstrated high-resolution cortical mapping. Multiple-subject level analysis using high-density electrocorticography yielded over 1300 electrode positions. **Conclusions:** Electrocorticography during deep brain stimulation is a valuable research method for movement disorders and, based on a moderate sized consecutive clinic sample, appears safe with risks no greater than those associated with DBS surgery itself.

## 1. Introduction

Electrocorticography (ECoG) during deep brain stimulation (DBS) presents a unique opportunity for intraoperative neuroscience research. DBS is a well-established surgical treatment for movement disorders, during which burr holes allow intracranial access for testing microelectrodes and/or macroelectrode lead placement. Through the same burr holes, subdural ECoG strip electrodes can be placed for localized cortical recordings [1], allowing for simultaneous collection of cortical ECoG signals, subcortical local field potentials (LFPs) and spiking neuronal activities. Real-time cortical and subcortical brain activity during behavior and stimulation can be captured, as patients remain awake during the procedure.

Clinical application of ECoG includes seizure detection and functional mapping of cortical areas during resective surgeries [2,3]. Its use in research has been expanding, especially in fundamental neuroscience brain mapping [4,5,6], and more recently in the development of brain–computer and brain–machine interface systems [7,8,9,10]. Increasingly, ECoG is used during DBS to study the cortical-basal ganglia network in movement disorders [11,12,13,14,15,16,17,18], provide insight into the underlying mechanisms for the therapeutic effects of DBS [19], and support the development of closed-loop stimulation systems for movement disorders [20,21,22,23].

Large-scale studies, including Sisterson et al. (200 patients) and Panov et al. (367 patients), have confirmed the technical aspects, safety, and utility of conducting research using ECoG during DBS surgery [24,25]. However, these studies had a limited spatial resolution of the electrophysiological data, and analyses were typically performed at the single-subject level.

In contrast, the present study employs high-density electrocorticography arrays with 63-contact grids and applies a standardized anatomical framework to enable multi-subject, group-level analysis in a common coordinate space. This approach allows for higher-resolution cortical mapping and improved integration of electrophysiological data across subjects. The aim of this study is therefore not only to demonstrate feasibility and safety, but also to highlight a methodological advancement in the acquisition and analysis of high-resolution intraoperative electrocorticography data in patients undergoing deep brain stimulation surgery. This work supports the growing role of intraoperative ECoG in studying movement disorders and contributes to the foundation for future research into adaptive, closed-loop neuromodulation systems.

## 2. Materials and Methods

### 2.1. Patient Selection and Consent

Patients considered for participation in the study were scheduled for DBS lead placement surgery for PD or ET in the “awake” state at the Johns Hopkins School of Medicine between February 2021 and September 2024. The exclusion criteria included inability to undergo awake DBS and any other exclusion as surmised by the multidisciplinary movement disorder surgery committee at Johns Hopkins (i.e., significant subcortical dementia or other surgically prohibitive co-morbidity). The Johns Hopkins Institutional Review Board approved the study. Patients were informed about the study during an in-person meeting with the neurosurgeon, and written informed consent was obtained.

### 2.2. Surgical Planning

A preoperative MRI was obtained and imported into the DBS planning system (Brainlab Elements, Brainlab AG, Munich, Germany). Based on the condition (PD, ET) the subcortical target (the subthalamic nucleus (STN) or ventral intermediate nucleus (VIm) of the thalamus, respectively) was selected. The entry point of the lead and the burr hole location was chosen solely based on clinical criteria, with consideration to avoid large vasculature, sulci, and the ventricular system. The ECoG target cortical coverage was marked on the cortical surface using the planning system. The cortical target area for PD and ET was the ‘hand knob’ of the primary motor cortex area inferred from the location of the precentral gyrus.

### 2.3. ECoG Placement

All recordings were performed during awake deep brain stimulation surgery under light IV dexmedetomidine sedation, minimizing anesthetic-related effects on cortical activity. Robotic stereotactic assistance was used (ROSA, Zimmer Biomet, Warsaw, IN or Globus ExcelsiusGPS, Globus Medical, Audubon, PA, USA). A Leksell stereotactic frame was placed on the patient’s head and used for head fixation during the surgery. After positioning, a 3D cone beam intraoperative CT image set was obtained (Medtronic O-arm, Medtronic, Minneapolis, MN, USA). This data was downloaded onto the robotic system and co-registered with a previously acquired head CT and MRI examination.

Following the robotic system registration process, the scalp was marked at the sites for the anticipated burr holes based on the DBS implant trajectory. The targeted cortical areas for ECoG strip placement were marked on the scalp. After skin incision and scalp dissection, a perforator was used to drill bilateral burr holes. The burr hole lead fixation system rings were then securely mounted. All patients underwent unilateral ECoG recordings based on the site of the initial DBS lead implant, which was typically the patient’s most symptomatic side. The dura was opened widely with a scalpel, and the dural leaflets were removed to prevent potential deflection of the DBS leads. The brain was gently depressed with a Penfield #3 spatula, and the subdural recording strip electrode was passed into the subdural space, aiming to center the electrode under the marked cortical site. The cabling was secured to the drapes with a clamp. The strip electrode cabling was then connected via the appropriate connection cables to the data acquisition system (Blackrock NSP, Blackrock Microsystems, Salt Lake City, UT, USA).

The insertion guide tube cannulae for microelectrode recordings and DBS electrode placement were inserted through the burr hole and the working arm of the robotic system, followed by the microelectrodes (Alpha Omega NeuroProbe, Alpha Omega Engineering, Nazareth Illit, Israel). Following placement of the microelectrodes, the burr hole was covered with gelfoam and fibrin glue to prevent CSF egress and minimize brain shift during the experimental recording sessions. Microelectrode recording was used to confirm a trajectory through the STN, and the microelectrode tip was stopped when the LFP recording electrode, at 3 mm above the tip electrode, was placed in the dorsolateral (motor) region of the STN. Stimulation testing using the mapping microelectrode system and stimulation through the DBS leads was used to localize the optimal target for lead placement. Following localization of the DBS target with the microelectrodes, the patient performed the experimental tasks, which took no more than 25 min. Behavioral tasks were administered in a consistent manner across patients, and recordings were obtained during comparable phases of the surgical procedure. Efforts were made to maintain stable recording conditions.

**Figure 1:**
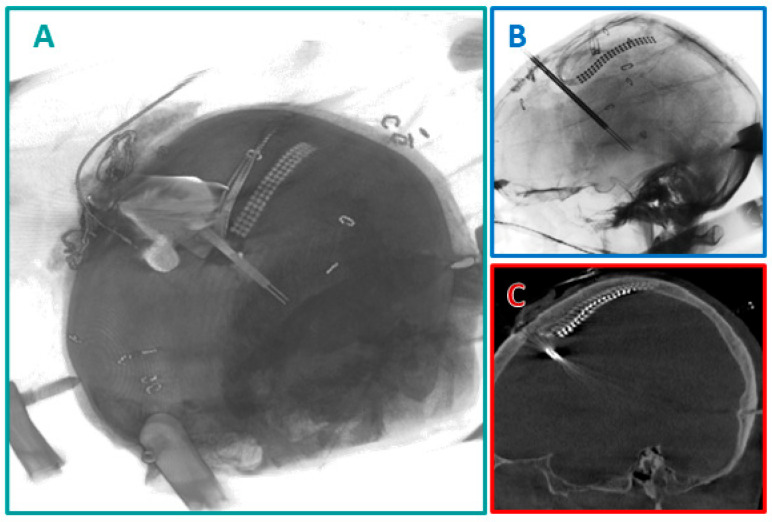
Intraoperative imaging showing the subdural placement of a 63-contact ECoG strip and the DBS leads at their target. (**A**) 3D reconstruction based on intraoperative CT, (**B**) Left-sided lateral fluoroscopy image (**C**), intraoperative CT (O-arm, Medtronic) image.

The placement of the subdural strip and microelectrodes was verified using the fluoroscopic mode of the O-arm and a 3D CT O-arm spin (Figure 1). Following this, the subdural strip electrode was removed and the DBS lead was placed and positioning was confirmed with macrostimulation mapping. Within 24 h of the surgery, a postoperative CT or MRI was obtained as part of routine care.

### 2.4. ECoG Characteristics

Subdural strip electrodes (PMT Corporation, Chanhassen, MN, USA) used for electrocorticography (ECoG) contained 63 numbered contacts arranged in a 21 × 7 grid configuration (21 rows, 7 columns). Each circular contact had a diameter of 1 mm with 3 mm center-to-center spacing. The overall electrode strip measured approximately 6.85 cm in length and 0.9 cm in width, with a thickness of 0.76 mm. Electrodes featured inline tail connections, 4 leads, and color-coded tails (blue, green, violet, white) for ease of channel identification.

### 2.5. Analysis of Anatomical and Electrophysiological Data

Analysis of anatomical and electrophysiological data on single and multiple subject levels was performed following the protocol by Stolk et al. [26]. Using the FieldTrip toolbox [27] for MATLAB (version 2023b, The Mathworks, Natick, MA, USA), preoperative T1-weighted MRI scans and postoperative CT scans were co-registered and electrode coordinates were aligned. This allowed for anatomical localization of the contacts in the anterior commissure posterior commissure (ACPC) coordinate system.

**Figure 2:**
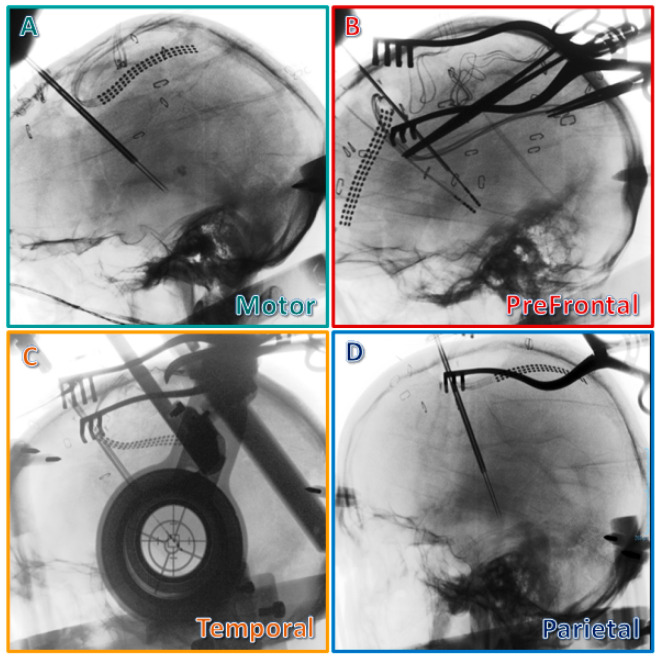
Fluoroscopic intraoperative images are displaying the subdural placement of a 63-contact ECoG strip while targeting various structures, including (**A**) Motor cortex, (**B**) Dorsolateral prefrontal cortex, (**C**) Superior temporal gyrus, and (**D**) Posterior parietal cortex.

Brain surface reconstruction using Freesurfer (version 7.4.1, Laboratory for Computational Neuroimaging, Charlestown, MA, USA, http://surfer.nmr.mgh.harvard.edu/ accessed on 15 March 2026) was performed based on the preoperative MRI. This allowed visualization of the subdural strip contacts in the ACPC coordinate system on the native brain surface anatomy. Figure 2 displays the sample cortical structures covered by strip electrodes, including the motor cortex, dorsolateral prefrontal cortex, superior temporal gyrus, and posterior parietal cortex. Following anatomical analysis, the neurophysiological data were processed and visualized on the exact reconstruction.

For the purpose of multiple-subject neurophysiological analysis, anatomical data of multiple subjects was integrated on a single normalized brain. ACPC contact coordinates for each subject were transformed to the Montreal Neurological Institute (MNI) coordinate space [26]. This was performed using a non-linear volume-based normalization procedure implemented in SPM (version 12, Functional Imaging Laboratory, London, UK, https://www.fil.ion.ucl.ac.uk/spm/ accessed on 15 March 2026), aligning individual anatomical MRI scans to the MNI template, after which contact coordinates were transformed accordingly. MNI contact coordinates for each subject were then plotted on a standardized MNI brain template, allowing for data integration.

### 2.6. Analysis of Postoperative Outcomes 

Routine postoperative CT scans, taken within the first 24 h after surgery, were examined for possible hemorrhage, infarcts, or other complications. Patients were monitored for at least 6 months postoperatively, and surgical complications (such as infections, chronic subdural hematoma, intraparenchymal hematoma, or venous infarction) were recorded in a database. Given the descriptive nature of the study, no formal statistical comparisons were performed.

## 3. Results

### 3.1. Patient Characteristics

A total of 36 patients were included in the study, with 26 undergoing DBS implantation for Parkinson’s disease (PD) and 10 for essential tremor (ET) (Table 1). In 34 cases, subdural ECoG strips were placed over the sensorimotor cortex using the same burr hole created for DBS electrode placement. In two patients, the strips were targeted to the dorsolateral prefrontal cortex for research purposes unrelated to motor physiology. In one case, strip placement did not result in adequate coverage of the primary motor cortex, specifically the hand knob area, and was excluded from group-level physiological analysis due to a lack of usable physiological signal.

### 3.2. Complication Rates and Details 

A total of four postoperative complications were observed, resulting in an overall surgical complication rate of 11.1% (Table 2). Three patients developed infections (8.3%), while one patient experienced a chronic subdural hematoma (2.8%). Of the infections, one was at the burr hole incision, two were at the superoauricular incision, and one occurred at the pulse generator site. The burr hole incision infection occurred ipsilateral to the side of ECoG strip placement.

The patient who developed a subdural hematoma presented with worsening headaches two months after surgery. Imaging confirmed a chronic hemorrhage on the hemisphere contralateral to the ECoG strip, and surgical drainage was performed without complication. At the latest follow-up, this patient had no lasting neurological deficits.

### 3.3. Example of Processing Anatomical and Physiological Data

Anatomical and neurophysiological data were analyzed at both the single-subject and group-subject levels. Electrode contact positions were localized using a combination of preoperative MRI and postoperative CT imaging, co-registered in ACPC space and transformed into MNI coordinates for cross-subject analysis.

Based on the applied pipeline, which includes rigid MRI–CT co-registration, subvoxel electrode localization, and correction for brain shift through projection to the cortical surface, localization error is expected to be on the order of a few millimeters. Prior studies using similar methodologies have demonstrated that such approaches can reduce electrode localization error to approximately 3 mm or less [26]. Accordingly, the spatial accuracy in the present study is expected to fall within this range.

**Figure 3:**
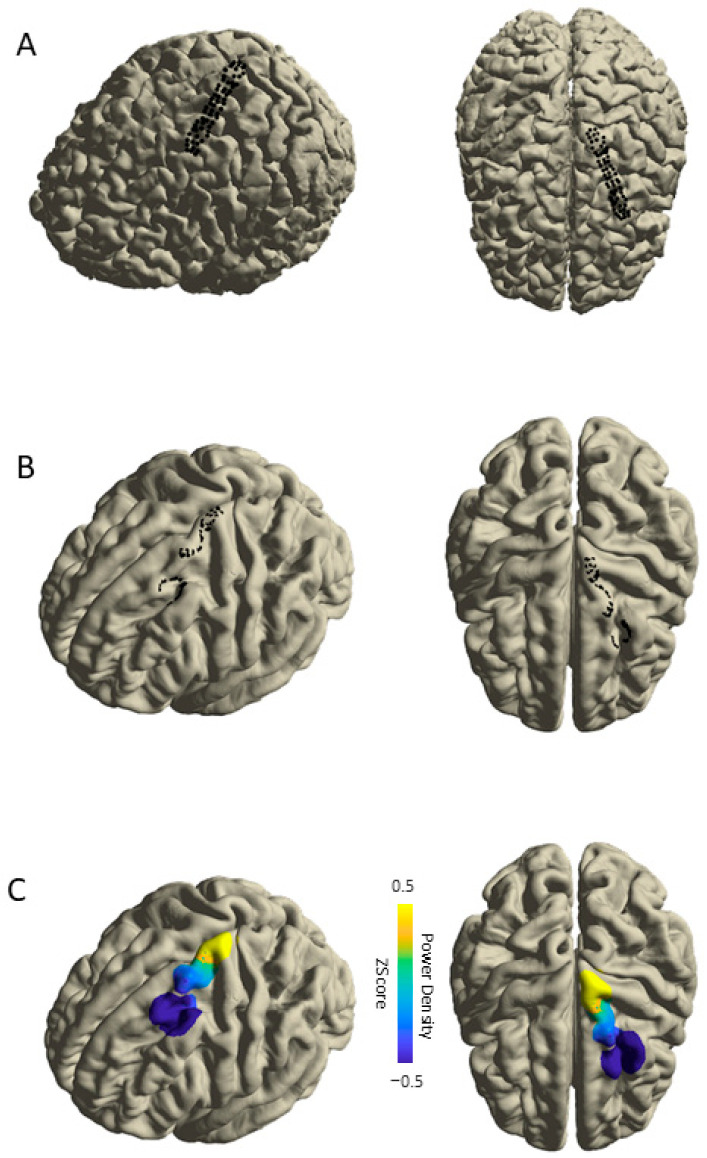
Single-subject analysis of anatomical and neurophysiological data. (**A**) Contact visualization on the native brain in the anterior and posterior commissures coordinate system. (**B**) Normalized contact positions visualization on an MNI brain reconstruction. (**C**) Neurophysiological data (beta frequency band power at rest) mapped onto the MNI brain.

Figure 3 illustrates a representative single-subject analysis. Electrode positions are shown on both the subject’s native brain surface in ACPC space (Figure 3A) and on a normalized brain in MNI space (Figure 3B). Corresponding neurophysiological data, resting-state beta power, are visualized in Figure 3C, demonstrating spatial variability in cortical activity across the grid.

**Figure 4:**
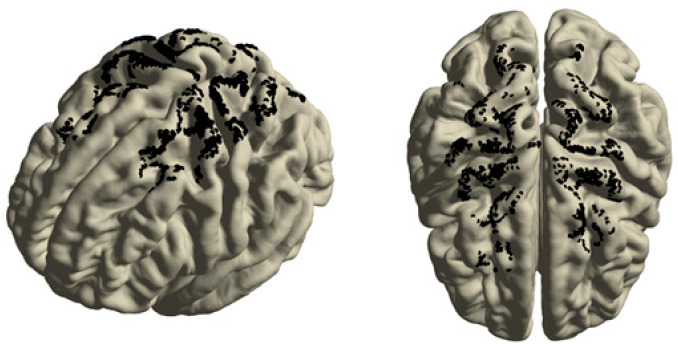
Multiple-subject anatomical analysis. MNI-space electrode localization on a normalized MNI-space brain for 21 subjects.

Group-level anatomical analysis was performed on a subset of 19 patients with STN-targeted implantation for PD. This included eight patients with left-sided and eleven with right-sided ECoG strips, each consisting of 63 contacts. Electrode positions from all subjects were projected onto a standardized MNI brain to generate a group anatomical map (Figure 4), allowing for the visualization of consistent sensorimotor coverage across the cohort.

**Figure 5:**
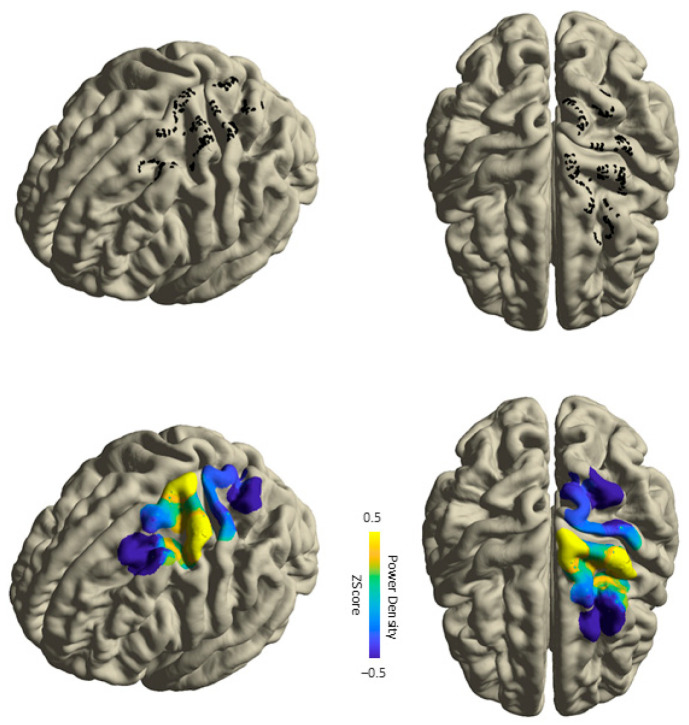
Multiple-subject analysis of anatomical and neurophysiological data. (**Upper**) MNI-space electrode localization on a normalized MNI-space brain for three subjects. (**Lower**) Neurophysiological data (beta frequency band power at rest) mapped onto the MNI brain for three subjects.

Figure 4 shows the integration of group-level anatomical and physiological data. Resting-state beta activity is displayed across multiple subjects on a common cortical surface, highlighting both the feasibility and utility of high-density ECoG in capturing detailed spatial patterns of oscillatory activity during DBS procedures. To illustrate the combination of anatomical localization and electrophysiological measurements, Figure 5 displays the locations of electrodes across the subjects in a normalized brain model. It also presents the activity in the beta frequency range, which is used to map the motor cortex.

**Figure 6:**
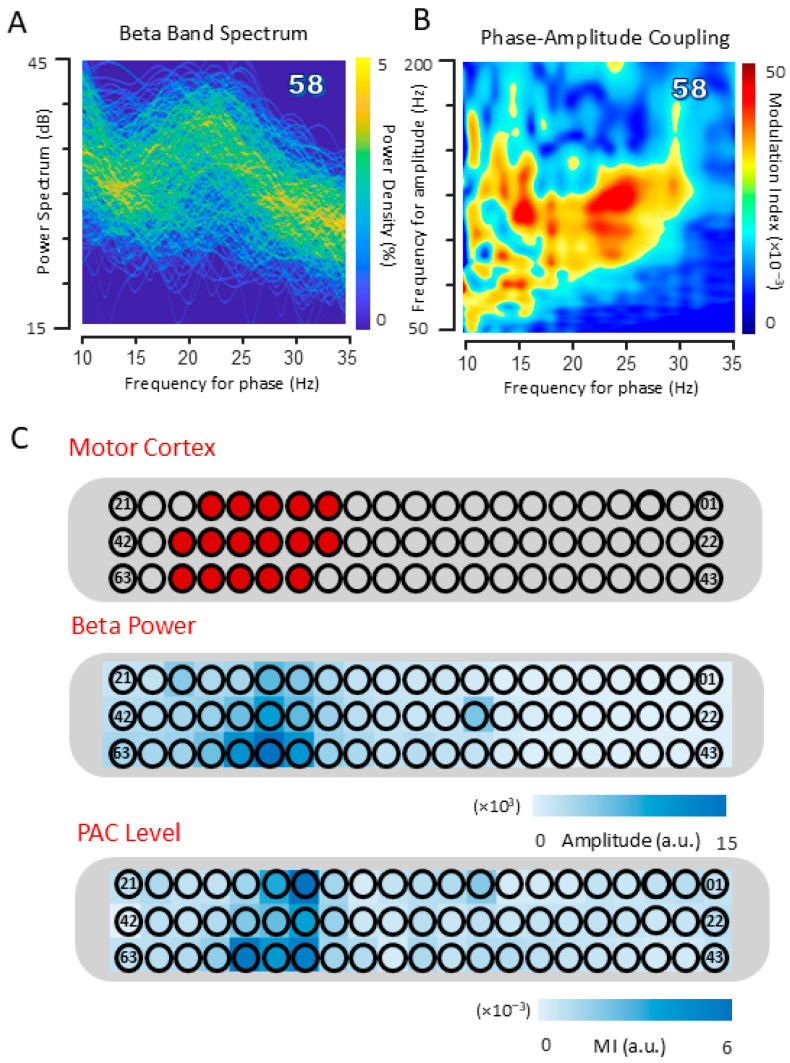
(**A**) The persistence spectrum of the beta band power at rest, which is a time–frequency view showing the percentage of time that beta oscillations are present in a motor cortex recording, is displayed. (**B**) Phase–amplitude coupling for the same ECoG signal from panel A is shown using the modulation index quantification method. (**C**) Mapping of the 63-contact ECoG strip electrode to the surface of the cortex is shown here, utilizing anatomical information, beta band power, and beta-gamma phase–amplitude coupling measurements.

After the electrodes are implanted in the designated target area, various intraoperative paradigms can be utilized during the surgical procedure of deep brain stimulation lead implantation. Taking advantage of this opportunity provides unique access to the human brain, allowing for the collection of valuable neurophysiological data. Figure 6 shows electrophysiological measurements from the motor cortex of the representative subject. The persistence spectrum of beta band power, which provides a time–frequency representation of the percentage of time that beta oscillations are present in the motor cortex of a Parkinson’s disease (PD) patient during intraoperative recordings at rest, is illustrated in Figure 6A. The figure shows that as the duration of beta oscillations in the motor cortex increases over time, the percentage of time they are present rises, resulting in a brighter or “hotter” appearance in the display. The phase–amplitude coupling of the ECoG signal from panel A is clearly demonstrated using the modulation index quantification method (Figure 6B). Mapping of the 63-contact ECoG strip electrode to the cortical surface is presented here, using anatomical information, beta band power, and beta-gamma phase–amplitude coupling measurements.

**Figure 7:**
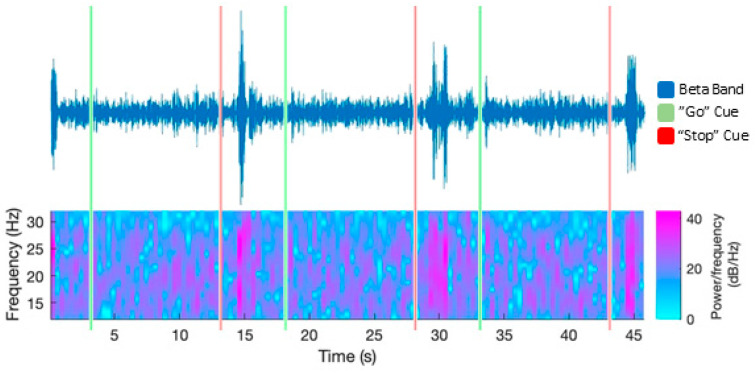
Beta band dynamics during an upper limb motor task. The beta band filtered signal (**top**) and time–frequency analysis (**bottom**) recorded during a repetitive grasping task from a subject with PD are shown. Green lines indicate when the subject was shown a visual cue to start moving, and red lines indicate when the subject was shown a visual cue to stop moving.

In addition to passive intraoperative recording, a variety of cognitive and motor-related paradigms can be conducted during surgical procedures. Figure 7 illustrates beta band dynamics during an upper limb motor task. The top section shows the beta band filtered signal, while the bottom section presents the time–frequency analysis recorded during a repetitive grasping task from a subject with PD. Green lines indicate when the subject received a visual cue to start moving, and red lines indicate when the subject received a visual cue to stop moving.

## 4. Discussion

### 4.1. Safety and Complications

As ECoG strip placement during DBS procedures is performed solely for research purposes and provides no direct therapeutic benefit, ensuring patient safety is essential. In comparison to a standard DBS procedure (without strip placement), the complication rate in this cohort was lower for each type of complication, including infections [28,29], subdural hemorrhages [32], intraparenchymal hemorrhages [30,31], and venous infarctions [32]. Two of three infections developed on sites unrelated to strip placement (not at the burr hole where the strip was placed); the superoauricular and pulse generator sites. However, one of the three infections occurred at the burr hole site, ipsilateral to the ECoG strip implantation, making the likelihood of a link between the infection and strip placement higher. Additionally, an indirect contribution through prolonged operative time cannot be entirely excluded. The chronic subdural hematoma developed on the hemisphere contralateral to the ECoG strip placement, making a causal relationship unlikely; it was successfully treated with surgical drainage, with no lasting neurological deficits. Overall, the complication rate in this cohort falls within the range reported in the literature for standard DBS procedures. These findings suggest that ECoG strip placement does not appear to increase the overall risk of complications and are consistent with previously published large-scale studies [24,25].

### 4.2. High-Resolution, Multi-Subject Data Integration

This report presents anatomical and neurophysiological data analyzed at both single and multiple subject levels. While prior large-scale studies have established the safety of intraoperative ECoG during deep brain stimulation, they have generally relied on lower-density electrode configurations and analyses performed at the single-subject level [24,25]. A major advantage of this approach is the ability to perform anatomically standardized, group-level analysis with high spatial resolution. In this cohort, 21 ECoG strips (63 contacts each) were registered to a single MNI brain (Figure 4), yielding a total of 1323 electrode positions and a broad cortical surface coverage. Combining high-density electrode arrays with a standardized anatomical pipeline advances intraoperative ECoG research by improving spatial precision and reproducibility. This approach supports detailed analysis of cortical activity during rest and intraoperative tasks and is well suited for large-scale studies of movement disorder physiology.

### 4.3. Research Opportunities and Potential Clinical Applications

Intraoperative ECoG during DBS procedures has been widely used to investigate the neurophysiology of movement disorders [16,19,33,34,35]. The burr hole created for DBS lead implantation provides access for ECoG strip placement, enabling cortical recordings from the surrounding surface area (Figure 5). The specific cortical region recorded depends on the movement disorder studied and the burr hole location [36]. Numerous studies have leveraged this approach to advance understanding of cortical dynamics in movement disorders [37,38,39,40,41,42,43].

Ongoing research in electrocorticography focuses on advancing closed-loop stimulation systems for movement disorders [20,21,22,23]. High-resolution, multi-subject ECoG analysis offers a standardized method for collecting and integrating cortical data, supporting the development of adaptive neuromodulation strategies. Such data may contribute to future research into adaptive, closed-loop neuromodulation systems and impact therapeutic approaches in care for patients with Parkinson’s disease and essential tremor.

### 4.4. Limitations

This study has several limitations that should be considered when interpreting the findings. First, the single-center design and relatively small sample size of 36 patients limit statistical sensitivity and the ability to detect small or moderate differences, particularly in subgroup analyses. No formal a priori power calculation was performed, as the study was designed as a feasibility and safety analysis. Additionally, the absence of a direct control group of patients undergoing DBS without ECoG limits conclusions regarding comparative surgical risk. Accordingly, outcomes are interpreted descriptively and contextualized with respect to previously published literature rather than through within-study inferential statistical comparisons. Future larger-scale, controlled, or multicenter studies will be required to validate these findings and enable adequately powered analyses.

Second, electrode localization accuracy was not directly quantified. However, the MRI–CT co-registration and normalization pipeline incorporates established methods for brain shift correction and electrode projection, with reported localization errors of approximately 3 mm. While the precise error in this dataset cannot be determined, it is expected to fall within this range. Future studies incorporating direct validation methods could further refine these estimates.

Intraoperative electrophysiological recordings are inherently subject to potential confounding factors, including patient fatigue, fluctuations in attention, and variability in surgical conditions. Although recordings were performed in awake patients under standardized conditions, these factors cannot be fully eliminated and may introduce variability in the recorded signals. At the same time, this reflects real-world intraoperative conditions and supports the feasibility of acquiring meaningful high-density electrocorticography data in a clinical setting.

Finally, inclusion of all electrode placements in the safety analysis introduces heterogeneity, as different cortical targets may carry varying anatomical and procedural risks. Differences in cortical accessibility, vascular anatomy, and proximity to critical structures may influence both the technical complexity of electrode placement and the likelihood of adverse events. Therefore, complication rates should be interpreted in the context of this variability.

## 5. Conclusions

This report demonstrates that high-density electrocorticography during awake DBS surgery is a feasible method for obtaining high-resolution cortical dataand, importantly, adds to the literature regarding the safety of this research method. Concerns have been raised regarding the invasiveness of ECoG placement for purely research purposes, but in this moderately sized cohort, it does not seem to be associated with an increased rate of complications compared to DBS surgery alone. By integrating anatomical localization across subjects using standardized coordinate systems, this approach enables group-level analyses with improved spatial resolution. This represents a methodological advancement over prior intraoperative ECoG studies and provides a foundation for future research into cortical physiology and the development of closed-loop neuromodulation systems.

## Figures and Tables

**Table 1:** Patient demographics and ECoG anatomical coverage.

Characteristic	PD	ET	Total
Number of patients	26	10	36
Median age (range) in years	66 (50–75)	68 (53–79)	67 (50–79)
No. of 9 contact strips placed	3	1	4
No. of 63 contact strips placed	23	9	32
No. of ECoG placements over sensorimotor cortex	24	10	34
No. of ECoG placements over the dorsolateral prefrontal cortex	2	0	2

**Table 2:** Postoperative complications.

Complication	No. of Cases	Reported Rate in the Literature
Infection	3 (8.3%)	Up to 9.9% [28,29]
Subdural hematoma	1 (2.8%)	3.0% [30]
Intraparenchymal hematoma	0 (0.0%)	0.6–2.9% [30,31]
Venous infarction	0 (0.0%)	0.8% [32]

## Data Availability

The data and code supporting the findings of this study are restricted due to participant privacy and will be available from the corresponding author upon reasonable request.

## Abbreviations

The following abbreviations are used in this manuscript:

ACPC	Anterior commissure posterior commissure
DBS	Deep brain stimulation
ECoG	Electrocorticography
ET	Essential tremor
IV	Intravenous
LFP	Local field potentials
MNI	Deep brain stimulation
PD	Parkinson’s disease
STN	Subthalamic nucleus
VIm	Ventral intermediate nucleus
